# Acinetobacter zhairhuonensis sp. nov., isolated from sediments of the East China Sea

**DOI:** 10.1099/ijsem.0.006936

**Published:** 2025-10-22

**Authors:** Qian Jiang, Yike Wang, Kaiwen Zheng, Yuangui Tang, Yunlu Cui, Qunjian Yin, Daoqiong Zheng, Jinzhong Xu, Daxiong Ji, Dongdong Zhang, Sanjit Chandra Debnath, Pinmei Wang

**Affiliations:** 1 Ocean College, Zhejiang University, Zhoushan 316021, Zhejiang, PR China; 2 Shenyang Institute of Automation, Chinese Academy of Sciences, Shenyang 110169, Liaoning, PR China; 3 College of Atmospheric Sciences, Sun Yat-sen University, Zhuhai 519082, Guangdong, PR China; 4 Key Laboratory of Tropical Marine Ecosystem and Bioresource, Fourth Institute of Oceanography, Ministry of Natural Resources, Beihai 536015, Guangxi, PR China; 5 Fourth Institute of Oceanography, Ministry of Natural Resources, Beihai 536015, Guangxi, PR China

**Keywords:** *Acinetobacter*, genome sequencing, marine sediments, polyphasic taxonomy

## Abstract

A novel aerobic bacterial strain, designated as *Acinetobacter zhairhuonensis* sp. nov. A7.4^T^, was isolated from East China Sea sediments near Zhairuo island. Cells were Gram-stain-negative, non-motile short rods, forming milky, moist colonies. Growth occurred at 10–35 °C, pH 6.0–9.0 and 0–5% NaCl concentrations (w/v). Strain A7.4^T^ was catalase-positive and oxidase-negative. Major fatty acids (>10%) included summed feature 3 (C_16:1_ ω7c/C_16 :1_ ω6c), C_18:1_ ω9c and C_16:0_. The polar lipid profile comprised diphosphatidylglycerol, phosphatidylglycerol, phosphatidylethanolamine, two phospholipids and two aminolipids. Ubiquinone Q-9 and Q-8 predominated. The genome (3.57 Mb, 41.3 mol% GC) included one chromosome and a plasmid. The core-genome phylogeny placed A7.4^T^ within *Acinetobacter*, forming a distinct clade with *Acinetobacter kanungonis* PS-1^T^, *Acinetobacter tibetensis* Y-23^T^ and *Acinetobacter tandoii* CIP107469^T^. Average nucleotide identity (ANI), average amino acid identity (AAI) and digital DNA–DNA hybridization (dDDH) values between strain A7.4^T^ and 87 validly published *Acinetobacter* species were below species thresholds (96% ANI/AAI, 70% dDDH). Phenotypic, genomic and chemotaxonomic data support the proposal of *Acinetobacter zhairhuonensis* sp. nov., with type strain A7.4^T^ (=MCCC 1K07162^T^=LMG 32567^T^).

The genus *Acinetobacter* was initially proposed by Brisou and Prevot [1], with the earliest recorded species dating to 1911, when Beijerinck isolated a soil-derived bacterium subsequently reclassified from *Micrococcus calcoaceticus* [2]. The genus *Acinetobacter* is taxonomically classified within the family *Moraxellaceae* (order *Pseudomonadales*, class *Gammaproteobacteria*). *Acinetobacter* species exhibit remarkable ecological adaptability, colonizing diverse habitats including soil [3,4], aquatic systems [5,6], organisms [7,8] and food products [9]. According to the List of Prokaryotic names with Standing in Nomenclature (July 2025 release), the genus currently recognizes 118 species, with 87 maintaining a validly published name under the International Code of Nomenclature of Prokaryotes. These Gram-stain-negative, non-motile bacteria exhibit oxidase-negative and catalase-positive characteristics, with certain strains displaying haemolytic activity and gelatin hydrolysis capabilities. The genome typically comprises a single circular chromosome (2.6–4.7 Mb) and a strain-dependent set of plasmid replicons [10]. During a marine microbial diversity investigation in the East China Sea, strain A7.4^T^ was isolated from marine sediments near Zhairuo Island, Zhejiang province. This isolate formed milky, circular colonies on mineral salt medium (MM) agar. Phenotypic and biochemical characterization, together with genomic analysis and comparisons, confirmed its membership in the genus *Acinetobacter*; nonetheless, it was shown to be distinct from all other *Acinetobacter* species. Based on the polyphasic taxonomic study, we propose *Acinetobacter zhairhuonensis* sp. nov. (type strain A7.4^T^=MCCC 1K07162^T^=LMG 32567^T^).

## Strain isolation

Strain A7.4^T^ was isolated from sediments of Zhairuo island (29° 56′ N, 121° 04′ E), Zhoushan city, Zhejiang province, China, in March 2019. One gram of beach sediments from the island was collected, suspended in artificial seawater, gradient diluted and spread on MM agar (1.0 g K_2_HPO_4_, 1.0 g KH_2_PO_4_, 2.0 g NaNO_3_, 0.5 g MgSO_4_.7H_2_O, 0.5 g (NH_4_)_2_SO_4_, 0.2 mg Na_2_MoO_4_ and 20.0 g agar per litre ddH_2_O, pH 7.0) [11]. A milky white colony with a diameter of 2 mm was picked and purified by the streak-plate technique on a fresh MM agar medium and designated as A7.4^T^. Strain A7.4^T^ has been deposited in the Marine Culture Collection of China (MCCC) and the Belgian Co-ordinated Collections of Micro-organisms (BCCM/LMG).

## Genomic characteristics

**Fig. 1.**
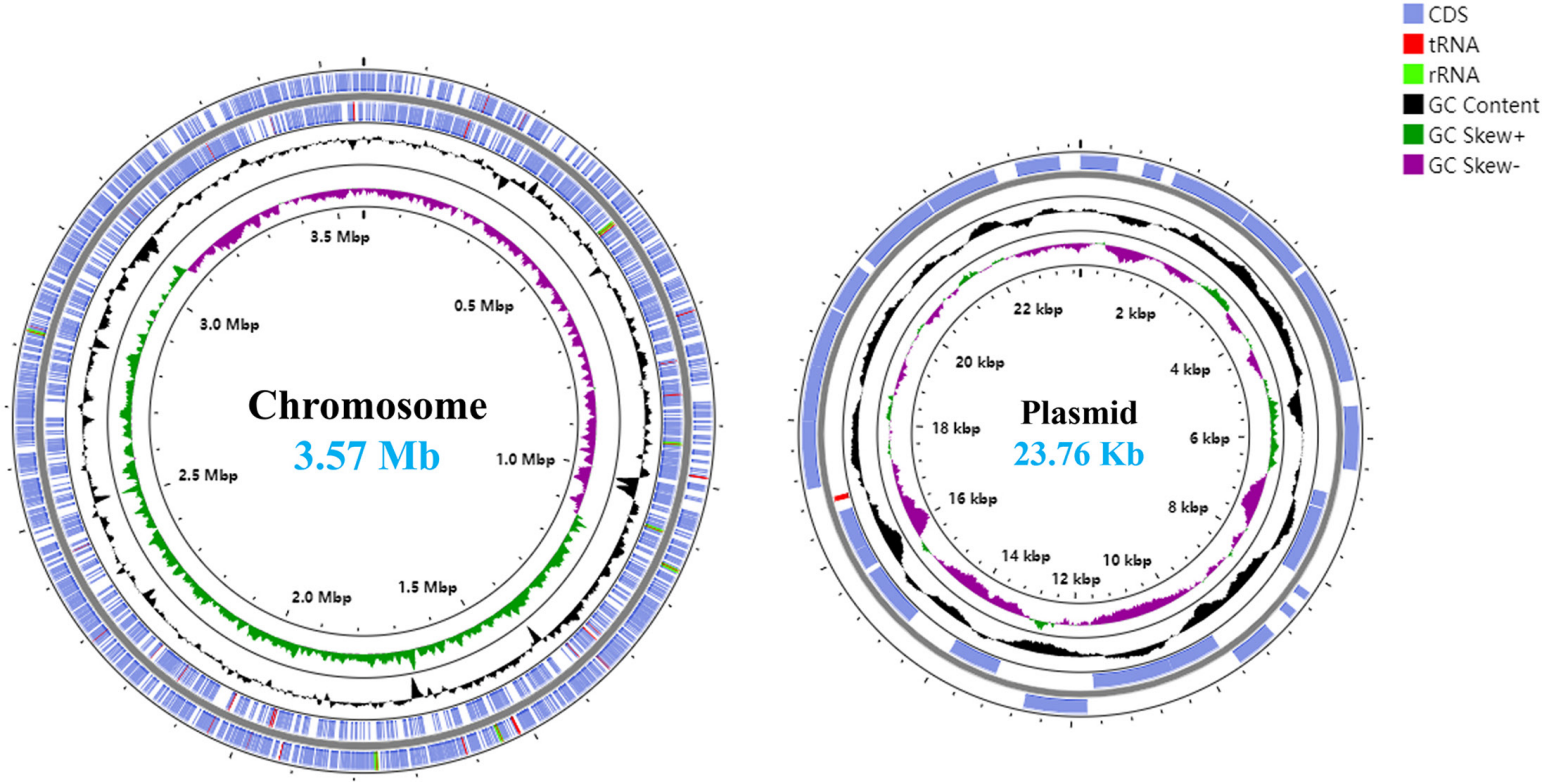
*A. zhairhuonensis* A7.4^T^ genome circular maps illustrated with the CGViewer pipeline, including one chromosome and one plasmid: CDS (blue), tRNA (red), rRNA (light green), G+C content (black), GC skew+ (dark green), GC skew- (violet).

The genomic DNA of A7.4^T^ was extracted using the MiniBEST Universal Genomic DNA Extraction Kit (Takara, Japan). The complete genome of strain A7.4^T^ was obtained using Illumina short-read sequencing and Nanopore long-read sequencing. Illumina paired-end sequencing (2×150 bp) was performed on a NovaSeq platform. Cutadapt was used to remove the adapters and bad quality reads [12]. Nanopore sequencing was conducted according to previously described methods [13,14]. Primary genome assembly was conducted with NECAT using Nanopore reads [15], followed by error correction with Pilon (v.1.13) using the high-quality Illumina reads [16]. The assembled genome was annotated using National Center for Biotechnology Information (NCBI) Prokaryotic Genome Annotation Pipeline (version 6.9) with default parameters [17]. Using nanopore sequencing and Illumina technology, an intact genome of A7.4^T^ was assembled, comprising a single circular chromosome and one plasmid (Fig. 1). The genome size of strain A7.4^T^ is 3.57 Mb. The genomic DNA G+C content of strain A7.4^T^ is 41.3 mol%, which is within the range (34.9–49.6 mol%) reported for members of *Acinetobacter* [10,18]. A total of 3,401 genes were predicted in the genome of strain A7.4^T^, including 3,245 protein-coding genes, 7 stretches of 5 S-16S-23S rRNA genes, 81 tRNAs, 4 ncRNAs and 50 pseudogenes.

## Genome-based taxonomy and phylogeny

**Fig. 2.**
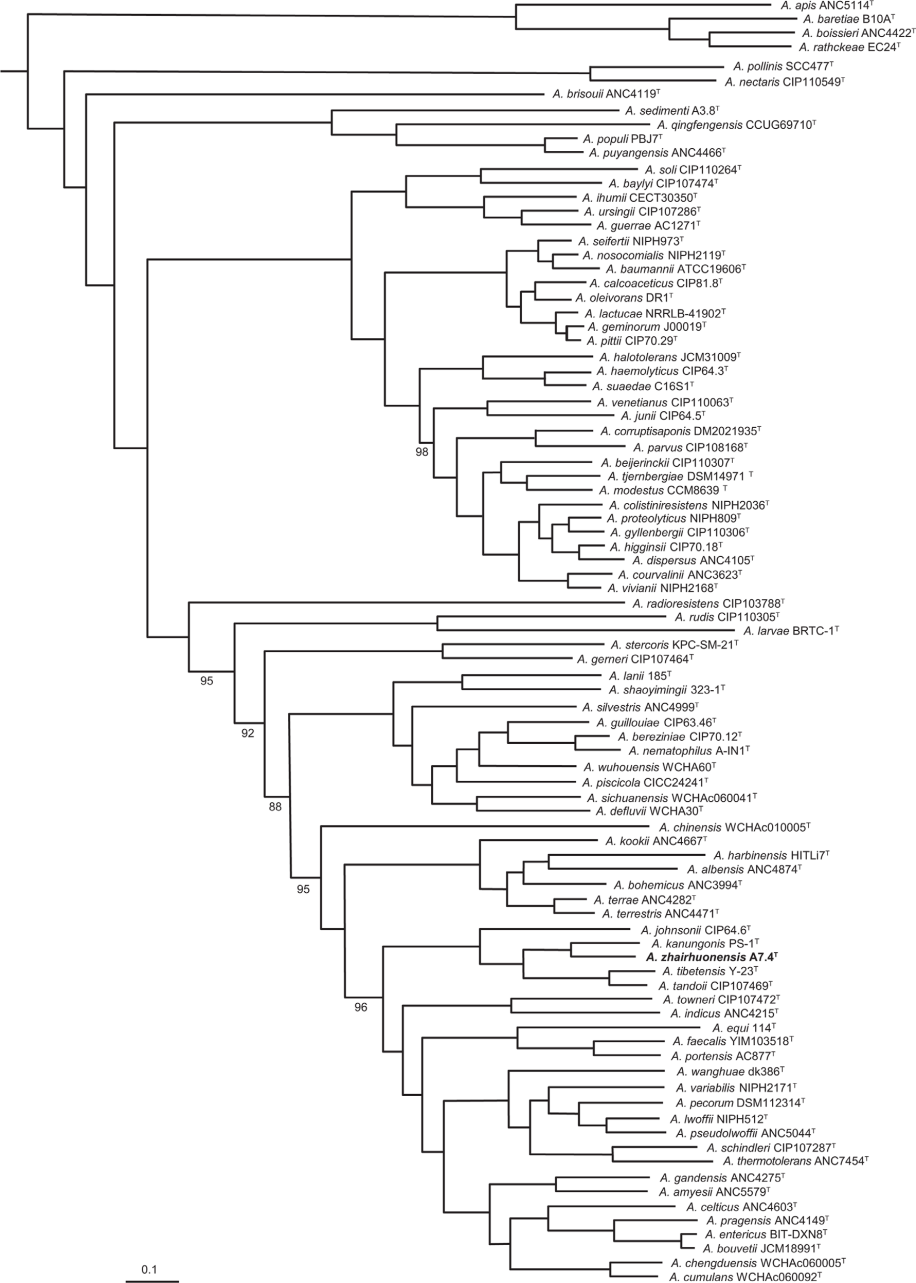
Phylogenetic tree reconstructed from 717 single-copy core genes shared between *A. zhairhuonensis* A7.4^T^ and 87 validly published species of the genus *Acinetobacter*. Bootstrap support values (percentage of 1,000 replicates) are indicated at nodes only when<100%. The position of strain A7.4^T^ is highlighted in bold.

Current 16S rRNA gene sequencing analyses demonstrate limited resolution for distinguishing closely related genomic species within *Acinetobacter* due to the gene’s low polymorphism [19]. Genus-wide core-genome phylogenetic analysis represents the current gold standard for determining phylogenetic positions of novel bacterial genomes, surpassing single-gene marker approaches [6,8, 20, 21]. Protein sequences of strain A7.4^T^ and 87 validly published *Acinetobacter* species were retrieved from NCBI (https://www.ncbi.nlm.nih.gov/), with OrthoFinder [22] identifying 717 single-copy orthologous genes (each showing >40% amino acid identity). Phylogenetic reconstruction was performed using FastTree [23] with visualization in Evolview v3 [24]. The core-genome phylogeny (Fig. 2) revealed A7.4^T^ forming a distinct subclade with *Acinetobacter kanungonis* PS-1^T^, which clustered with a sister branch containing *Acinetobacter tibetensis* Y-23^T^ and *Acinetobacter tandoli* CIP107469^T^. These three phylogenetically proximate strains were consequently selected as references for polyphasic characterization of A7.4^T^.

The complete 16S rRNA gene sequences of strain A7.4^T^ and other validly published species were aligned by the clustalw software [25]. Phylogenetic trees were constructed using neighbour-joining and maximum evolution methods of the mega-x program [26] with 1,000 replicates to yield the bootstrap values. Phylogenetic analysis based on 16S rDNA (Figs S1 and S2, available in the online Supplementary Material) confirmed A7.4^T^ within the *Acinetobacter* genus, forming a separate evolutionary branch with *A. kanungonis* PS-1^T^, but failed to cluster with *A. tibetensis* Y-23^T^ and *A. tandoli* CIP107469^T^, diverging from the core-genome phylogeny results.

Genomic comparisons using average nucleotide identity (ANI), digital DNA–DNA hybridization (dDDH) and average amino acid identity (AAI) consistently supported the novel species status of strain A7.4^T^ (Table S1). Comparative analyses between strain A7.4^T^ and all 87 validly published *Acinetobacter* species demonstrated ANI values (calculated using JSpeciesWS [27]) ranged from 78.3% to 95.1%, dDDH values (Genome-to-Genome Distance Calculator (GGDC) v.3.0 formula 2 [28]) varied between 21.4% and 68.9% and AAI values (EzAAI [29]) spanned 84.5% to 94.98% – all below respective species delineation thresholds (ANI/AAI<96% [8,30]; dDDH <70% [28,30]). Among these, the three phylogenetically closest strains (*A. kanungonis*, * A. tibetensis* and *Acinetobacter tandoii*) exhibited the highest genomic similarity values (ANI, 84.17–89.8%; dDDH, 28.3–40.0%; AAI, 90.78–94.98%), yet still showed divergence exceeding species delineation criteria. These results confirm strain A7.4^T^ as a novel species and are consistent with the core-genome phylogeny, further validating the superior accuracy of core-genome phylogenetic analysis compared to 16S rRNA gene-based phylogenetic approaches.

## Screening of publicly available metagenomic datasets and NCBI databases

To further evaluate the occurrence and abundance of strain A7.4^T^ in natural environments, particularly marine habitats, blast analyses were performed targeting four genetic markers of strain A7.4^T^. Given that 16S rRNA V4 and V3–V4 hypervariable regions are widely used in microbiota studies due to their taxonomic accuracy [31], these regions were screened across 4,663 metagenomic samples (439 finished; 4,224 draft) from marine environments (ocean, 230; marine, 2,544; sea, 1,450) in the Integrated Microbial Genomes and Microbiomes [32]. High-coverage, high-identity (>97%) matches were further assessed using full-length 16S rRNA sequences and the *rpoB* gene region (RNA polymerase *β*-subunit; positions 2,916–3,773) [21]. The 16S rRNA V4 region yielded high-coverage, high-identity (>99%) matches, whereas the V3–V4 region, full-length 16S rRNA and *rpoB* gene exhibited lower coverage and identity, with *rpoB* showing no significant hits or identities below 92% (Excel File Dataset S1).

The four genetic markers were additionally screened against NCBI databases (Excel File Dataset S1). blast searches of the 16S rRNA V4 region revealed numerous 100% coverage/100% identity matches, including seawater metagenomes (KDVZ00000000.1) and uncultured clones. The V3–V4 region similarly showed multiple hits with 100% coverage and >99% identity from uncultured clones, suggesting widespread occurrence of A.7.4^T^-related sequences. For full-length 16S rRNA, high matches included *Acinetobacter* sp. ANC 4910 (SJNT01000006.1; 100% coverage, 99.0% identity) and an uncultured *Acinetobacter* sp. clone (HM159980.1; 100% coverage, 99.13% identity), in addition to A.7.4^T^ itself and the close relative *A. tandoii* (99.2% identity). However, genome-based comparisons (ANI, dDDH and AAI) between *Acinetobacter* sp. ANC 4910 and A.7.4^T^ indicated distinct species status, with all values below established thresholds (Table S1). No significant *rpoB* matches were observed (all identities<80%).

In summary, while the 16S rRNA V4 region confirms the environmental prevalence of strain A.7.4^T^, its identification based on a longer sequence remains limited. Obtaining additional isolates or high-quality metagenomes for this taxon requires further extensive investigation.

## Comparative genomics and functional profiling

The complete genome sequences of strain A7.4^T^ and 87 validly published *Acinetobacter* species were subjected to comprehensive *in silico* functional analysis, revealing both conserved metabolic features and strain-specific functional characteristics.

Metabolic pathway annotation using KofamKOALA [33] and KEGG Decoder [21] identified 116 functional pathways classified into 20 categories (Fig. S3). Consistent with other *Acinetobacter* species [21], strain A7.4^T^ is capable of synthesizing most essential amino acids and possesses PGA synthesis proteins for poly-*β*-1,6-*N*-acetylglucosamine production, a polymer critical for biofilm formation [34]. Respiratory energy metabolism is supported by the presence of F-type ATPase and cytochrome bd complexes, correlating with the aerobic growth requirements of strain A7.4^T^. Genomic evidence of non-motility, indicated by absent flagellar genes, was confirmed through transmission electron microscopy (Fig. S4) and motility assays. Notably, while metabolic pathway profiles showed high conservation between strain A7.4^T^ and its phylogenetically proximate relatives (*A. kanungonis*, *A. tibetensis* and *A. tandoii*), significant genomic divergence was evident through ANI (<96%) and dDDH (<70%) values. This conservation of core metabolic functions despite genomic differentiation parallels observations in other *Acinetobacter* species descriptions [21].

Secondary metabolite gene clusters were investigated using the antiSMASH bacterial version website [35] with default parameters. The heatmap was created using an online platform (https://www.bioinformatics.com.cn) [36]. Comparative analysis of secondary metabolite biosynthetic gene cluster (BGC) profiles reveals distinct features in strain A7.4^T^ relative to other members of the genus (Fig. S5). While strain A7.4^T^ exhibits an intermediate overall BGC count (total=8), specific enrichments highlight its biosynthetic specialization. Notably, it encodes two non-ribosomal peptide synthetase (NRPS) clusters compared to phylogenetically close relatives such as *A. tibetensis*, *A. tandoii* and *A. kanungonis*, and more *β*-lactone BGCs than most *Acinetobacter* species. Conversely, strain A7.4^T^ lacks BGCs associated with hybrid (e.g. Polyketide Synthase-Nonribosomal Peptide Synthetase (PKS-NRPS)) and other undefined categories. Overall, strain A7.4^T^ combines moderate BGC diversity with specialized enrichments, distinguishing it from closely related species.

## Phenotypic traits

Cell morphology and size of strain A7.4^T^ were observed under light microscopy (E100; Nikon) and transmission electron microscopy (JEM1230, Hitachi). Gram reaction was examined with a commercial Gram staining kit (BD) following the manufacturer’s protocol. The phenotypic and physicochemical features of A7.4^T^ and its reference strains were tested under the same conditions. Gliding motility was tested with freshly cultured cells according to the hanging drop method, and motility was investigated by semi-solid Luria-Bertani (LB) agar () [37]. Anaerobic growth was tested on LB agar after incubating in an anaerobic box (AnaeroPack^TM^-Anaero; Mitsubishi Gas Chemical Co., Inc.) at 30 ℃ for 7 days according to the manufacturer’s instructions. After 24 h of incubation on LB agar medium at 30 ℃, colonies of strain A7.4^T^ were milky, round and moist with a diameter of ~2 mm. Transmission electron microscopy showed a short rod cell of ~1.0–1.3 µm×1.0–1.6 µm (Fig. S4). Strain A7.4^T^ was Gram-stain-negative, aerobic and non-motile.

OD_600_ was used to monitor the growth of strain A7.4^T^ in LB medium at different temperatures (10, 30, 35, 37 and 40 ℃), different NaCl concentrations (w/v, 0–10% at increments of 1%) and different pH (pH 4–10, at intervals of 1 pH unit). The bacterial growth was evaluated using the following media: LB agar, nutrient agar (NA), trypticase soy agar (TSA) and Reasoner’s 2A agar (R2A) (all from Oxoid), with incubation at 30 °C for 3 days. Strain A7.4^T^ grew on all of the tested media, with the fastest growth on LB agar medium. Strain A7.4^T^ grew at 10–35 ℃ and pH 6.0–9.0 with 0–5% (w/v) NaCl. The optimal growth was observed at 30 ℃, pH 7.0, and in the presence of 2.5% (w/v) NaCl.

Oxidase and catalase activities were investigated by colour change in 1% (w/v) tetramethyl-*p*-phenylenediamine and generation of bubbles in 3.0% (v/v) H_2_O_2_, respectively. Methyl red and Voges–Proskauer reaction, H_2_S production, starch hydrolysis, gelatin hydrolysis, lipase (Tweens 20/40/60/80), phenylalanine deaminase and DNA hydrolysis were determined according to previously described methods [18]. Tests for the assimilation of the 36 carbon sources were performed according to previously described methods [38]. Other biochemical properties were tested using API 20NE and API 50CH tests (bioMérieux) according to the manufacturer’s instructions.

H_2_S production, catalase, Tweens 20, 40, 60, 80 and tyrosine hydrolysis tests of strain A7.4^T^ are positive. Hydrolysis of starch, casein, acidification of d-glucose, haemolysis of sheep blood, liquefaction of gelatin, methyl red, Voges–Proskauer, phenylalanine deaminase and DNA hydrolysis tests are negative. In the API 20NE kit, a positive reaction for aesculin hydrolysis and assimilation of malate, citrate and phenylacetate is observed. The key and differential characteristics between strain A7.4^T^ and three reference strains are given in Table 1. The negative reaction of strain A7.4^T^ on API kits is shown in Table S2. The shared carbon source assimilation patterns between strain A7.4^T^ and reference strains are listed in Table S3.

**Table 1.** Key and differential characteristics of strain A7.4^T^ and reference strains

Characteristic*	1†	2	3	4
Growth at 37 (℃)	−	+	+	+
Growth at 35 (℃)	+	+	+	+
Growth at 30 (℃)	+	+	+	+
Acidification of d-glucose	−	−	−	−
Haemolysis of sheep blood	−	−	−	−
Liquefaction of gelatin	−	−	−	−
H_2_S production	+	−	+	−
API 20NE				
Esculin hydrolysis	+	+	−	−
Caprate assimilation	+	+	+	+
Malate assimilation	+	+	+	+
Citrate assimilation	+	−	+	+
Phenylactate assimilation	+	+	+	+
Assimilation (growth on)‡				
*trans*-Aconitate	+	−	+	+
*β*-Alanine	−	−	−	+
2,3-Butanediol	+	+	+	−
Glutarate	w	−	−	+
l-Leucine	−	−	+	+
Putrescine	+	−	+	+
l-Tartrate	−	−	+	+
Tricarballylate	+	−	+	+
Tryptamine	+	+	−	+

*+, Positive; −, negative; w, weakly positive.

†Strains: 1, *A. zhairhuonensis* A7.4T; 2, *A. tibetensis* Y-23T; 3, *A. tandoii* CIP107469T; 4, *A. kanungonis* PS-1T.

‡Assimilation data were provided by Professor Alexandr Nemec (National Institute of Public Health, Prague, Czech Republic), and all other data were generated in this study.

## Chemotaxonomic traits

The fatty acid [39] composition of the cells grown on the TSA medium for 2 days was determined according to the protocol of the Sherlock Microbial Identification System (MIDI) [40]. The predominant fatty acids of strain A7.4^T^ (> 5.0% of the total amounts) were summed feature 3 (C_16:1_ ω7c/C_16:1_ ω6c) (31.2%), C_18:1_ ω9c (22.2%), C_16:0_ (16.7%), C_12:0_ (7.5%) and C_12:0_ 3-OH (5.1%). The fatty acid composition of A7.4^T^ is consistent with previous results for recognized species of the genus *Acinetobacter*, justifying its placement in this genus [4,8]. The detailed fatty acid profile of strain A7.4^T^ is summarized in Table S4.

Polar lipids [41] were separated and analysed with TLC Silica gel 60 F_254_ (Merck 1.05554.0001) and a freshly prepared developing agent according to the method described by Minnikin *et al*. [42]. The polar lipids of strain A7.4^T^ contained diphosphatidylglycerol, phosphatidylglycerol, phosphatidylethanolamine, two phospholipids (PL1–2) and two aminolipids (Fig. S6). To determine respiratory quinones, strain A7.4^T^ was incubated in 500 ml LB medium for 2 days at 30 °C, harvested and lyophilized. Then respiratory quinones of strain A7.4^T^ were extracted and analysed by HPLC according to the process described previously [43]. The main quinones of strain A7.4^T^ are Q-8 and Q-9 (Fig. S7). The chemotaxonomic characteristics of strain A7.4^T^ compared with the closely related type strains *A. tibetensis* Y-23^T^ [11], *A. tandoii* CIP107469^T^ [44] and *A. kanungonis* PS-1^T^ [8] further support that strain A7.4^T^ represents a member of the genus *Acinetobacter*.

## Description of *Acinetobacter zhairhuonensis* sp. nov.

*Acinetobacter zhairhuonensis* (zhai.rhu.o.nen’sis. N.L. masc. adj. zhairhuonensis, pertaining to Zhairuo, an island of the East China Sea, from where the type strain was isolated).

Cells are Gram-stain-negative, aerobic, non-motile, oxidase-negative, catalase-positive, short rods, 1.0–1.3 µm wide and 1.0–1.6 µm long. It can grow in LB, NA, TSA and R2A medium, with LB yielding the fastest and best growth. After culturing on LB agar plates for 3 days, the colonies are round, smooth, milky, opaque and moist with a diameter of about 2 mm. Growth occurs at 10–35 ℃ (optimum 30 ℃), pH 6.0–9.0 (optimum pH 7.0) and 0–5% (w/v) NaCl (optimum 2.5%). Positive for H_2_S production, catalase, Tweens 20, 40, 60, 80 and tyrosine hydrolysis. Negative for hydrolysis of starch, casein, acidification of d-glucose, haemolysis of sheep blood, liquefaction of gelatine, methyl red, Voges–Proskauer, phenylalanine deaminase and DNA hydrolysis. Of 36 tested carbon sources, assimilation was positive for 22 substrates: acetate, *trans*-aconitate, 4-aminobutyrate, l-arginine, l-aspartate, benzoate, 2,3-butanediol, citrate, ethanol, l-glutamate, glutarate, l-histidine, 4-hydroxybenzoate, dl-lactate, d-malate, malonate, l-ornithine, phenylacetate, l-phenylalanine, putrescine, tricarballylate, tryptamine, whereas negative results were obtained for adipate, *β*-alanine, l-arabinose, azelate, citraconate, gentisate, d-gluconate, d-glucose, histamine, l-leucine, levulinate, d-ribose, l-tartrate and trigonelline. In the API 20NE kit, a positive reaction for aesculin hydrolysis and assimilation of caprate, malate, citrate and phenylacetate. All the other tests are negative in the API 50CH kit.

The predominant fatty acids include Summed feature 3 (C_16:1_ ω7c/C_16:1_ ω6c), C_18:1_ ω9c, C_16:0_, C_12:0_ and C_12:0_ 3-OH. Polar lipids include diphosphatidylglycerol, phosphatidylglycerol, phosphatidylethanolamine, two phospholipids (PL1–2) and two aminolipids. The main respiratory quinones are Q-8 and Q-9. The genome comprises a single circular chromosome and one plasmid. The genome size and genomic DNA G+C content are 3.57 Mb and 41.3 mol%, respectively.

The type strain, A7.4^T^ (=MCCC 1K07162^T^=LMG 32567^T^), was isolated from sediments of Zhairuo Island in Zhejiang, China. The GenBank accession numbers for the genome and 16S rRNA gene sequence of *A. zhairhuonensis* A7.4^T^ are GCA_047300875 and MW287273, respectively.

## Supplementary material

10.1099/ijsem.0.006936 Uncited Supplementary Material 1.

10.1099/ijsem.0.006936 Uncited Supplementary Material 2.

## Abbreviations

AAI: average amino acid identity
ANI: average nucleotide identity
BGC: biosynthetic gene cluster
dDDH: digital DNA–DNA hybridization
GGDC: Genome-to-Genome Distance Calculator
LB: Luria-Bertani
MM: mineral salt medium
NA: nutrient agar
NCBI: National Center for Biotechnology Information
NRPS: non-ribosomal peptide synthetase
PKS-NRPS: Polyketide Synthase-Nonribosomal Peptide Synthetase
R2A: Reasoner’s 2A agar
TSA: trypticase soy agar

## References

[1] Brisou J, Prevot AR (1954). Studies on bacterial taxonomy. X. The revision of species under *Acromobacter* group. Ann Inst Pasteur.

[2] Beijerinck M (1910). Koninklijke Nederlandse Akademie van Wetenschappen Proceedings Series B Physical Sciences.

[3] Choi JY, Ko G, Jheong W, Huys G, Seifert H (2013). *Acinetobacter kookii* sp. nov., isolated from soil. Int J Syst Evol Microbiol.

[4] Pan H, Li J, Liu H-H, Lu X-Y, Zhang Y-F (2023). *Acinetobacter tibetensis* sp. nov., isolated from a soil under a greenhouse in Tibet. Curr Microbiol.

[5] Li W, Zhang D, Huang X, Qin W (2014). *Acinetobacter harbinensis* sp. nov., isolated from river water. Int J Syst Evol Micr.

[6] Nemec A, Radolfová-Křížová L, Maixnerová M, Nemec M, Španělová P (2021). Delineation of a novel environmental phylogroup of the genus *Acinetobacter* encompassing *Acinetobacter terrae* sp. nov., *Acinetobacter terrestris* sp. nov. and three other tentative species. Syst Appl Microbiol.

[7] Wolf S, Barth-Jakschic E, Birkle K, Bader B, Marschal M (2021). *Acinetobacter geminorum* sp. nov., isolated from human throat swabs. Int J Syst Evol Microbiol.

[8] Das L, Deb S, Das SK (2021). Description of *Acinetobacter kanungonis* sp. nov., based on phylogenomic analysis. Int J Syst Evol Microbiol.

[9] Marí-Almirall M, Cosgaya C, Pons MJ, Nemec A, Ochoa TJ (2019). Pathogenic *Acinetobacter* species including the novel *Acinetobacter dijkshoorniae* recovered from market meat in Peru. Int J Food Microbiol.

[10] Nemec A, Whitman WB (2022). Bergey’s Manual of Systematics of Archaea and Bacteria.

[11] Zheng K, Hong Y, Guo Z, Debnath SC, Yan C (2022). *Acinetobacter sedimenti* sp. nov., isolated from beach sediment. Int J Syst Evol Microbiol.

[12] Martin M (2011). Cutadapt removes adapter sequences from high-throughput sequencing reads. EMBnet J.

[13] Qi L, Sui Y, Tang X-X, McGinty RJ, Liang X-Z (2023). Shuffling the yeast genome using CRISPR/Cas9-generated DSBs that target the transposable Ty1 elements. PLoS Genet.

[14] Yin J, He M, Liu X-X, Ren C-B, Liu H-H (2024). *Peteryoungia algae* sp. nov. isolated from seaweeds of Gouqi Island, China, and its unique genetic features among *Peteryoungia* strains. *Antonie van Leeuwenhoek*.

[15] Chen Y, Nie F, Xie S-Q, Zheng Y-F, Dai Q (2021). Efficient assembly of nanopore reads via highly accurate and intact error correction. Nat Commun.

[16] Walker BJ, Abeel T, Shea T, Priest M, Abouelliel A (2014). Pilon: an integrated tool for comprehensive microbial variant detection and genome assembly improvement. PLoS One.

[17] Tatusova T, DiCuccio M, Badretdin A, Chetvernin V, Nawrocki EP (2016). NCBI prokaryotic genome annotation pipeline. Nucleic Acids Res.

[18] Chen X-M, An D-F, He S-R, Yang S-J, Yang Z-Z (2023). *Acinetobacter faecalis* sp. nov., isolated from elephant faeces. Curr Microbiol.

[19] La Scola B, Gundi VAKB, Khamis A, Raoult D (2006). Sequencing of the rpoB gene and flanking spacers for molecular identification of *Acinetobacter* species. J Clin Microbiol.

[20] Nemec A, Radolfová-Křížová L, Maixnerová M, Nemec M, Shestivska V (2022). *Acinetobacter amyesii* sp. nov., widespread in the soil and water environment and animals. Int J Syst Evol Microbiol.

[21] Shestivska V, Španělová P, Krůtová M, Maixnerová M, Thiago Dobbler P (2024). Proposal of *Acinetobacter thermotolerans* sp. nov. to accommodate bovine feces-dwelling bacteria growing at 47 °C. Syst Appl Microbiol.

[22] Emms DM, Kelly S (2019). OrthoFinder: phylogenetic orthology inference for comparative genomics. Genome Biol.

[23] Price MN, Dehal PS, Arkin AP (2009). FastTree: computing large minimum evolution trees with profiles instead of a distance matrix. Mol Biol Evol.

[24] Subramanian B, Gao SH, Lercher MJ, Hu SN, Chen WH (2019). Evolview v3: a webserver for visualization, annotation, and management of phylogenetic trees. Nucleic Acids Res.

[25] Larkin MA, Blackshields G, Brown NP, Chenna R, McGettigan PA (2007). Clustal W and Clustal X version 2.0. *Bioinformatics*.

[26] Kumar S, Stecher G, Li M, Knyaz C, Tamura K (2018). MEGA X: molecular evolutionary genetics analysis across computing platforms. Mol Biol Evol.

[27] Richter M, Rosselló-Móra R, Oliver Glöckner F, Peplies J (2016). JSpeciesWS: a web server for prokaryotic species circumscription based on pairwise genome comparison. Bioinformatics.

[28] Meier-Kolthoff JP, Auch AF, Klenk HP, Göker M (2013). Genome sequence-based species delimitation with confidence intervals and improved distance functions. *BMC Bioinformatics*.

[29] Kim D, Park S, Chun J (2021). Introducing EzAAI: a pipeline for high throughput calculations of prokaryotic average amino acid identity. J Microbiol.

[30] Riesco R, Trujillo ME (2024). Update on the proposed minimal standards for the use of genome data for the taxonomy of prokaryotes. Int J Syst Evol Microbiol.

[31] Onywera H, Meiring TL (2020). Comparative analyses of Ion Torrent V4 and Illumina V3-V4 16S rRNA gene metabarcoding methods for characterization of cervical microbiota: taxonomic and functional profiling. Sci Afr.

[32] Chen I-MA, Chu K, Palaniappan K, Ratner A, Huang J (2023). The IMG/M data management and analysis system v.7: content updates and new features. Nucleic Acids Res.

[33] Aramaki T, Blanc-Mathieu R, Endo H, Ohkubo K, Kanehisa M (2020). KofamKOALA: KEGG ortholog assignment based on profile HMM and adaptive score threshold. Bioinformatics.

[34] Choi AHK, Slamti L, Avci FY, Pier GB, Maira-Litrán T (2009). The pgaABCD locus of *Acinetobacter baumannii* encodes the production of poly-beta-1-6-N-acetylglucosamine, which is critical for biofilm formation. J Bacteriol.

[35] Blin K, Shaw S, Augustijn HE, Reitz ZL, Biermann F (2023). antiSMASH 7.0: new and improved predictions for detection, regulation, chemical structures and visualisation. Nucleic Acids Res.

[36] Tang D, Chen M, Huang X, Zhang G, Zeng L (2023). SRplot: a free online platform for data visualization and graphing. PLoS One.

[37] Debnath SC, Chen C, Liu S-X, Di Y-N, Zheng D-Q (2019). *Flavobacterium sharifuzzamanii* sp. nov., isolated from the sediments of the East China Sea. Curr Microbiol.

[38] Nemec A, Musílek M, Maixnerová M, De Baere T, van der Reijden TJK (2009). *Acinetobacter beijerinckii* sp. nov. and *Acinetobacter gyllenbergii* sp. nov., haemolytic organisms isolated from humans. Int J Syst Evol Microbiol.

[39] Kämpfer P, Kroppenstedt RM (1996). Numerical analysis of fatty acid patterns of coryneform bacteria and related taxa. Can J Microbiol.

[40] Sasser M (1990). MIDI Technical Note 101.

[41] Komagata K, Suzuki K (1988). 4 lipid and cell-wall analysis in bacterial systematics. Method Microbiol.

[42] Minnikin DE, O’Donnell AG, Goodfellow M, Alderson G, Athalye M (1984). An integrated procedure for the extraction of bacterial isoprenoid quinones and polar lipids. J Microbiol Methods.

[43] Collins MD, Jones D (1981). Distribution of isoprenoid quinone structural types in bacteria and their taxonomic implication. Microbiol Rev.

[44] Carr EL, Kämpfer P, Patel BKC, Gürtler V, Seviour RJ (2003). Seven novel species of *Acinetobacter* isolated from activated sludge. Int J Syst Evol Microbiol.

