# Supplementary material for: Acinetobacter zhairhuonensis sp. nov., isolated from sediments of the East China Sea
Source: Int J Syst Evol Microbiol. 2025 Oct 22;75(10):006936. doi: 10.1099/ijsem.0.006936 (PMC12544349; doi:10.1099/ijsem.0.006936)
Supplement: Uncited Supplementary Material 1. [file ijsem-75-06936-s001.pdf]

Supplementary Information

Acinetobacter zhairhuonensis sp. nov., isolated from sediments of the East China Sea

Qian Jiang[1], Yike Wang[1], Kaiwen Zheng[1], Yuangui Tang[2], Yunlu Cui[3], Qunjian Yin[4,5], Daoqiong Zheng[1], Jinzhong Xu[1], Daxiong Ji[1], Dongdong Zhang[1], Sanjit Chandra Debnath[1], Pinmei Wang[1*]

[1] Ocean College, Zhejiang University, Zhoushan 316021, Zhejiang, P.R. China

[2] Shenyang Institute of Automation, Chinese Academy of Sciences, Shenyang 110169, Liaoning, P.R. China

[3] College of Atmospheric Sciences, Sun Yat-sen University, Zhuhai 519082, Guangdong, P.R. China

[4] Key Laboratory of Tropical Marine Ecosystem and Bioresource, Fourth Institute of Oceanography, Ministry of Natural Resources, Beihai 536015, Guangxi, P.R. China

[5] Fourth Institute of Oceanography, Ministry of Natural Resources, Beihai 536015, Guangxi, P.R. China

* Corresponding Authors: Pinmei Wang, wangpinmei@zju.edu.cn, ORCID: 0000-0003-2960-8573

Fig. S1

Neighbor-joining phylogenetic tree based on 16S rRNA gene sequences, revealing the relationship between strain A7.4^T and 87 validly published species of the genus *Acinetobacter*. Bootstrap values, calculated from 1000 replicates, are displayed at the nodes as percentage, with only those ≥ 50% shown at the branch points. *Agitococcus lubricus* DSM5822^T was used as an outgroup. The scale bar represents 0.01 substitutions per nucleotide position. The position of strain A7.4^T is highlighted in **bold**.

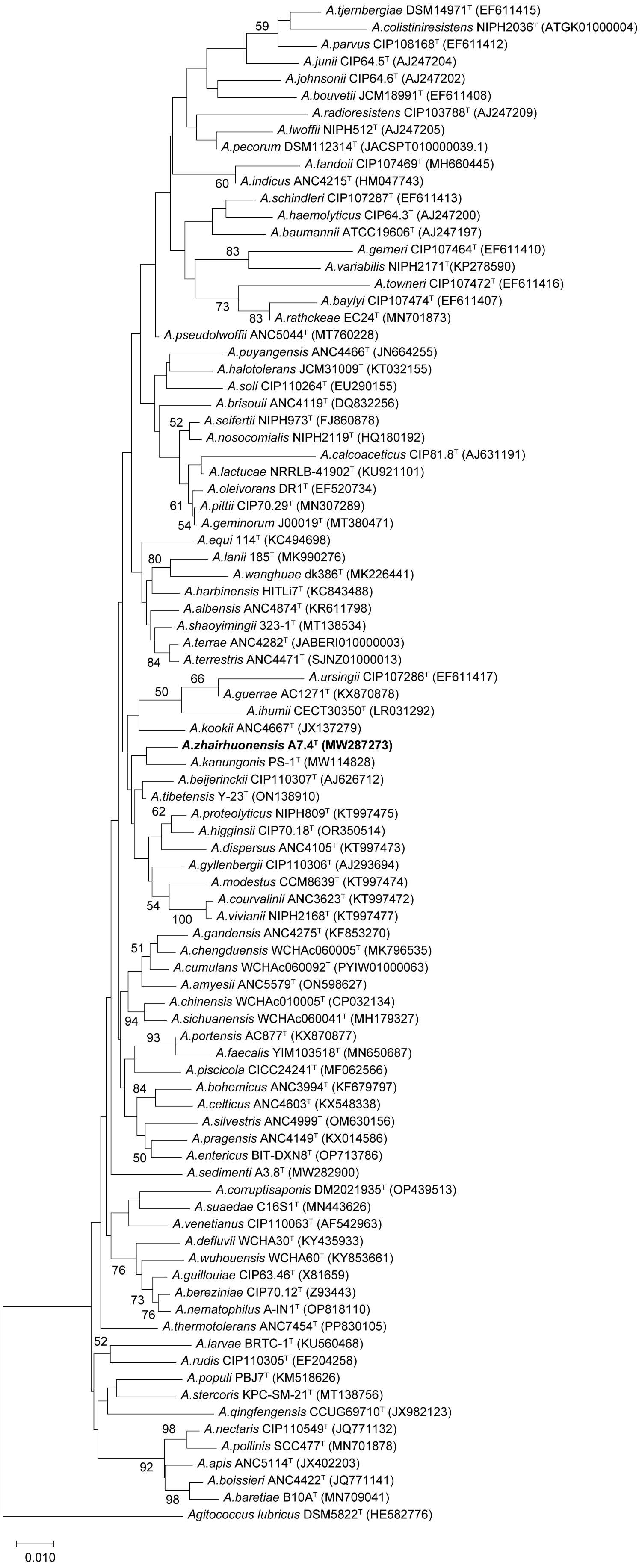

Fig. S2

Maximum-evolution phylogenetic tree based on 16S rRNA gene sequences, revealing the relationship between strain A7.4^{T} and 87 validly published species of the genus *Acinetobacter*. Bootstrap values, calculated from 1000 replicates, are displayed at the nodes as percentage, with only those ≥ 50% shown at the branch points. *Agitococcus lubricus* DSM5822^{T} was used as an outgroup. The scale bar represents 0.01 substitutions per nucleotide position. The position of strain A7.4^{T} is highlighted in **bold**.

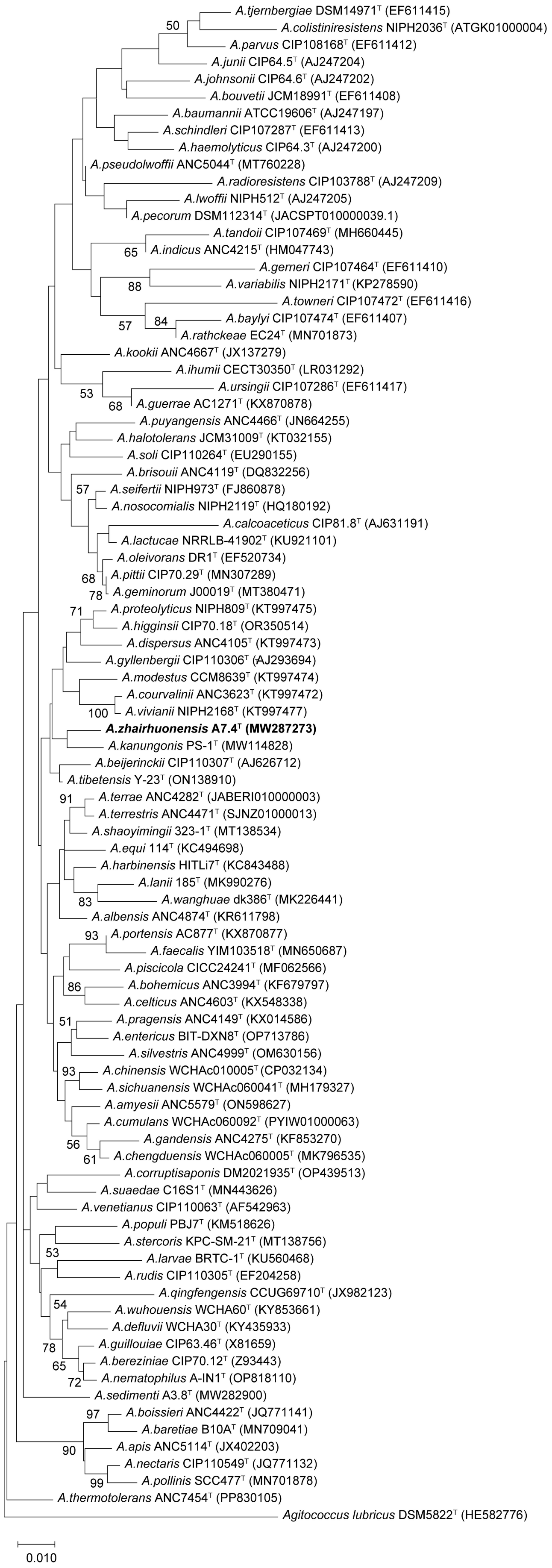

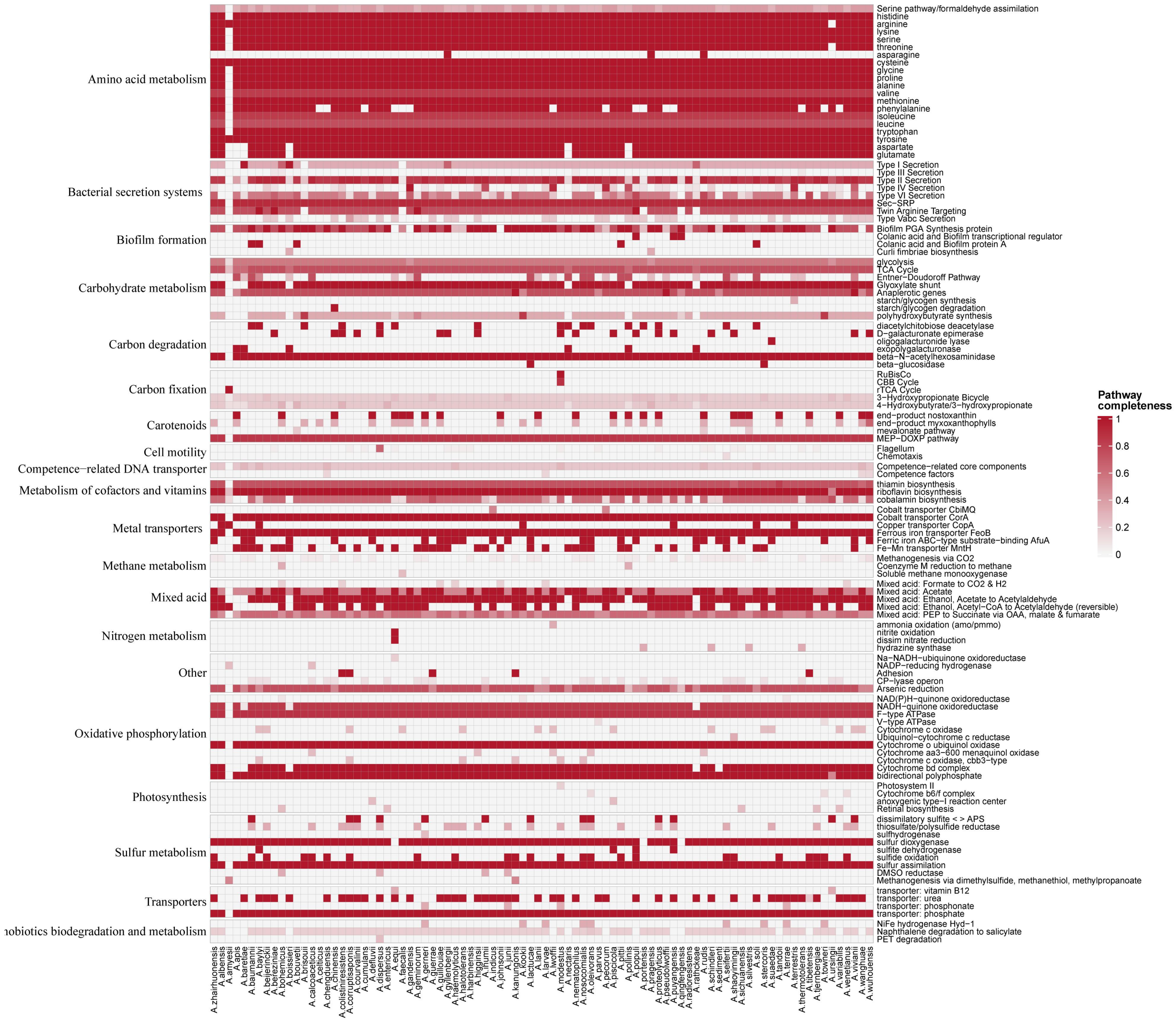

Fig. S3 Comparative metabolic profile analysis of *Acinetobacter zhairhuonensis* (first column) and type strains of 87 validly published *Acinetobacter* species.

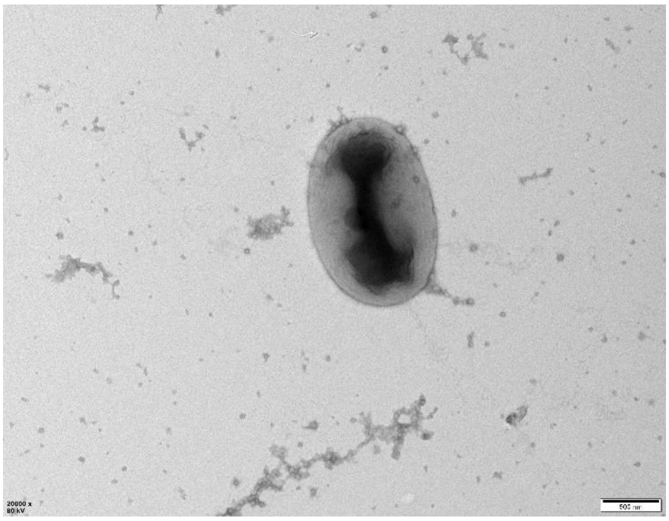

Fig. S4 Cell morphology of strain A7.4^T observed under transmission electron microscopy. The cells are short rod-shaped. Bar, 500 nm.

Fig. S5 Comparative analysis of secondary metabolite biosynthetic gene clusters (BGCs) in strain A7.4^{T} and 87 validly published *Acinetobacter* species. The heatmap depicts the distribution of various BGC types across the analyzed strains, with cell color intensity representing the relative abundance of each BGC category. PKS, Polyketide synthase; NRPS, Non-ribosomal peptide synthetase; RiPP, ribosomally synthesized and post-translationally modified peptide; NI-siderophore, NRPS-independent siderophores; Hybrid, a gene cluster with no less than 2 core genes, such as PKS-NRPS; Others, other undefined categories. The position of strain A7.4^{T} is highlighted in **bold**.

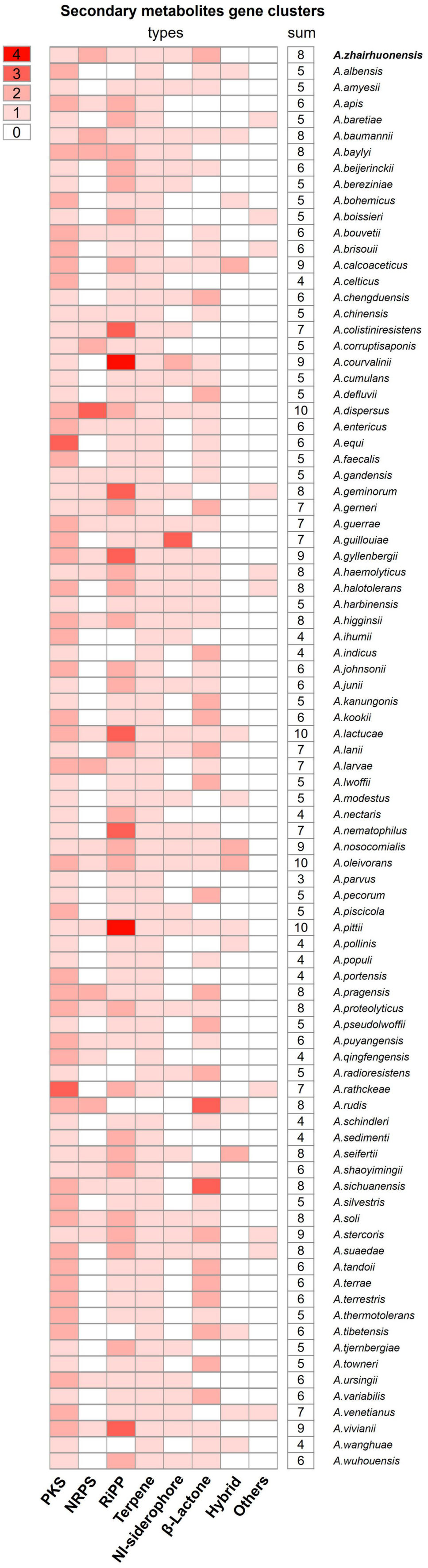

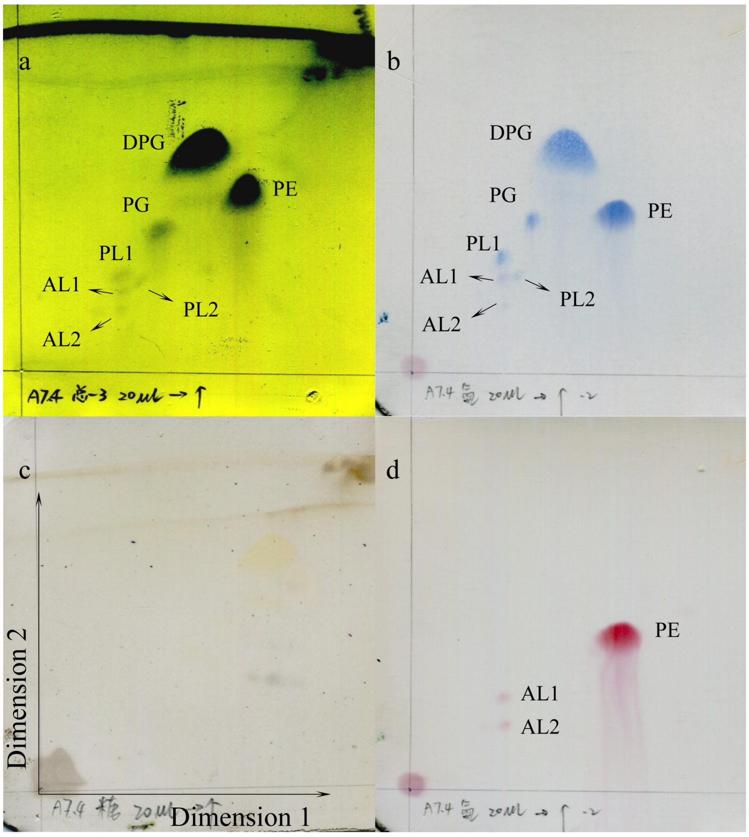

Fig. S6 Two-dimensional thin-layer chromatograms of the polar lipids from strain A7.4^T (a) Total polar lipids; (b) Phospholipids; (c) Glycolipids; (d) Aminolipids. Abbreviations: DPG, diphosphatidylglycerol; PE, phosphatidylethanolamine; PG, phosphatidylglycerol; PL1, PL2, phospholipid; AL1, AL2, aminolipid.

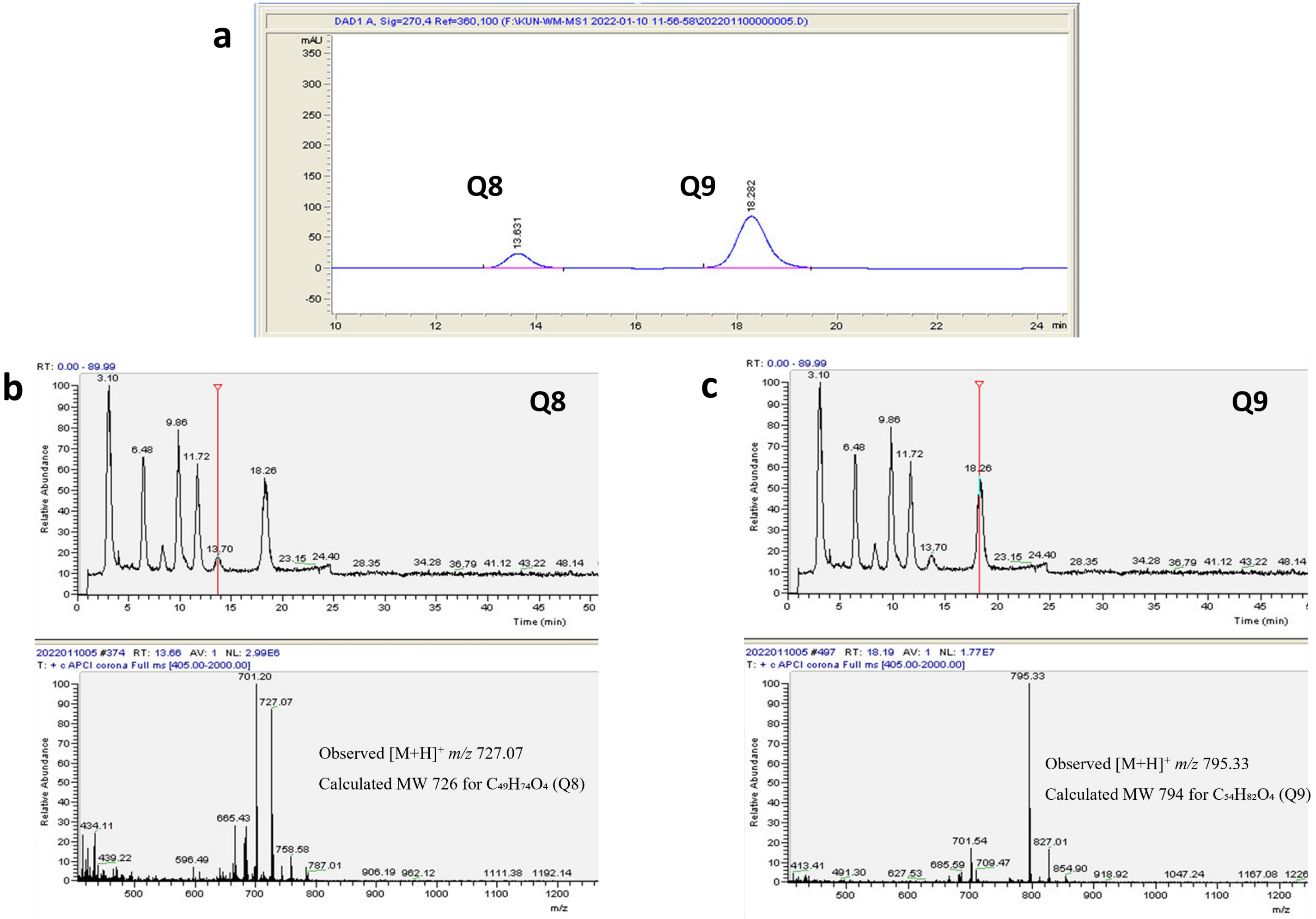

Fig. S7 HPLC (a) and HPLC-MS profiles of the respiratory quinones Q-8 (b) and Q-9 (c) in strain A7.4^T.

Table S1 Genomic relatedness values (ANI, dDDH, AAI) between strain A7.4^T and: (i) type strains of 87 validly published *Acinetobacter* species, and (ii) the closest match strain ANC4910

No.	Correct name	Sequenced strain	Genome acc.no.	ANI (%)	dDDH (%)	AAI（%）
1	*A.albensis*	ANC4874^T	FMBK00000000.1	75.84	20.7	79.42
2	*A.amyesii*	ANC5579^T	GCF_023499985.1	75.65	21.3	78.42
3	*A.apis*	ANC5114^T	FZLN00000000.1	71.74	21.3	71.32
4	*A.baretiae*	B10A^T	VTDM00000000.1	71.57	21.4	70.80
5	*A.baumannii*	ATCC19606^T	CP046654.1	74.30	21.0	76.94
6	*A.baylyi*	CIP107474^T	APPT00000000.1	73.92	20.9	76.04
7	*A.beijerinckii*	CIP110307^T	APQL00000000.1	74.41	21.0	76.49
8	*A.bereziniae*	CIP70.12^T	APQG00000000.1	74.84	21.8	76.60
9	*A.bohemicus*	ANC3994^T	APOH00000000.1	76.60	21.6	79.85
10	*A.boissieri*	ANC4422^T	FMYL00000000.1	71.63	20.9	70.87
11	*A.bouvetii*	JCM18991^T	AP024595.1	75.08	21.0	78.74
12	*A.brisouii*	ANC4119^T	APPR00000000.1	74.13	22.0	75.06
13	*A.calcoaceticus*	CIP81.8^T	APQI00000000.1	74.37	21.0	76.97
14	*A.celticus*	ANC4603^T	MBDL00000000.1	75.89	21.0	78.68
15	*A.chengduensis*	WCHAc060005^T	RCHC00000000.1	75.43	21.5	77.80
16	*A.chinensis*	WCHAc010005^T	CP032134.1	74.81	20.9	78.35
17	*A.colistiniresistens*	NIPH2036^T	ATGK00000000.1	74.49	21.0	76.42
18	*A.corruptisaponis*	DM2021935^T	CP125669.1	74.13	21.6	76.34
19	*A.courvalinii*	ANC3623^T	APSA00000000.1	74.35	21.6	76.64
20	*A.cumulans*	WCHAc060092^T	CP035934.2	75.50	21.8	77.79
21	*A.defluvii*	WCHA30^T	CP029397.2	75.24	22.0	77.44
22	*A.dispersus*	ANC4105^T	APRL00000000.1	74.51	21.2	76.34
23	*A.entericus*	BIT-DXN8^T	GCA_026168575.1	75.03	20.8	78.41
24	*A.equi*	114^T	CP012808.1	75.28	22.1	77.64
25	*A.faecalis*	YIM103518^T	WLYL00000000.1	75.79	21.1	78.81
26	*A.gandensis*	ANC4275^T	LZDS00000000.1	75.80	21.4	78.77
27	*A.geminorum*	J00019^T	JABELE000000000.1	74.37	21.2	76.98
28	*A.gerneri*	CIP107464^T	APPN00000000.1	74.57	21.9	76.31
29	*A.guerrae*	AC1271^T	LXGN00000000.1	74.07	20.4	76.18
30	*A.guillouiae*	CIP63.46^T	APOS00000000.1	75.14	21.8	76.74
31	*A.gyllenbergii*	CIP110306^T	ATGG00000000.1	74.57	21.1	76.31
32	*A.haemolyticus*	CIP64.3^T	APQQ00000000.1	74.29	21.7	76.70
33	*A.halotolerans*	JCM31009^T	SGIM00000000.1	74.25	20.9	76.47
34	*A.harbinensis*	HITLi7^T	JXBK00000000.1	75.38	20.5	78.40
35	*A.higginsii*	CIP70.18^T	GCA_000369525.1	74.68	21.5	76.28
36	*A.ihumii*	CECT30350^T	UYYC00000000.1	74.12	20.6	76.58
37	*A.indicus*	ANC4215^T	ATGH00000000.1	75.61	20.8	79.80
38	*A.johnsonii*	CIP64.6^T	APON00000000.1	76.45	22.0	79.82
39	*A.junii*	CIP64.5^T	APPX00000000.1	74.31	21.2	76.86
40	*A.kanungonis*	PS-1^T	JAACKC000000000.1	89.80	40.0	94.98
41	*A.kookii*	ANC4667^T	FMYO00000000.1	76.33	21.2	80.31
42	*A.lactucae*	NRRLB-41902^T	LRPE00000000.1	74.36	21.2	77.14
43	*A.lanii*	185^T	CP049916.1	75.13	21.7	77.02
44	*A.larvae*	BRTC-1^T	CP016895.1	72.87	21.9	73.20
45	*A.lwoffii*	NIPH512^T	AYHO00000000.1	75.13	21.0	78.22
46	*A.modestus*	CCM8639^T	BMDV00000000.1	74.61	21.7	76.64
47	*A.nectaris*	CIP110549^T	AYER00000000.1	71.97	21.5	72.01
48	*A.nematophilus*	A-IN1^T	JAPKMY01	74.73	21.1	76.75
49	*A.nosocomialis*	NIPH2119^T	APOP00000000.1	74.39	21.3	76.99
50	*A.oleivorans*	DR1^T	CP002080.1	74.33	21.1	76.86
51	*A.parvus*	CIP108168^T	APOM00000000.1	74.67	21.5	77.71
52	*A.pecorum*	DSM112314^T	GCA_014837015.1	74.92	20.6	78.09
53	*A.piscicola*	CICC24241^T	NIFO00000000.1	75.24	21.3	77.29
54	*A.pittii*	CIP70.29^T	APQP00000000.1	74.43	21.2	77.21
55	*A.pollinis*	SCC477^T	VTDQ00000000.1	71.76	21.2	71.79
56	*A.populi*	PBJ7^T	NEXX00000000.1	71.96	21.3	70.97
57	*A.portensis*	AC877^T	LWRV00000000.1	75.94	21.3	78.60
58	*A.pragensis*	ANC4149^T	LUAW00000000.1	74.97	20.6	78.70
59	*A.proteolyticus*	NIPH809^T	APOI00000000.1	74.64	21.5	76.31
60	*A.pseudolwoffii*	ANC5044^T	PHRG00000000.1	75.30	20.7	78.51
61	*A.puyangensis*	ANC4466^T	OANT00000000.1	71.80	21.4	70.88
62	*A.qingfengensis*	CCUG69710^T	VXKN00000000.1	71.59	21.1	70.59
63	*A.radioresistens*	CIP103788^T	APQF00000000.1	73.41	19.9	77.06
64	*A.rathckeae*	EC24^T	VTDO00000000.1	71.85	21.1	71.34
65	*A.rudis*	CIP110305^T	ATGI00000000.1	73.44	21.5	74.63
66	*A.schindleri*	CIP107287^T	APPQ00000000.1	74.88	21.1	77.92
67	*A.sedimenti*	A3.8^T	JAKUML000000000	72.29	23.4	70.48
68	*A.seifertii*	NIPH973^T	APOO00000000.1	74.60	21.4	77.00
69	*A.shaoyimingii*	323-1^T	CP049801.1	75.03	22.3	76.85
70	*A.sichuanensis*	WCHAc060041^T	PYIX00000000.2	75.24	21.7	76.90
71	*A.silvestris*	ANC4999^T	NEGB00000000.1	75.4	21.1	77.57
72	*A.soli*	CIP110264^T	APPU00000000.1	73.51	20.6	76.16
73	*A.stercoris*	KPC-SM-21^T	OOGT00000000.1	73.97	20.5	76.24
74	*A.suaedae*	C16S1^T	CP043909.1	74.20	21.4	76.81
75	*A.tandoii*	CIP107469^T	AQFM00000000.1	84.33	28.4	91.24
76	*A.terrae*	ANC4282^T	JABERI000000000.1	76.64	21.3	80.32
77	*A.terrestris*	ANC4471^T	SJNZ00000000.1	76.53	21.2	80.31
78	*A.thermotolerans*	ANC7454^T	CP156810.1	74.98	20.7	78.37
79	*A.tibetensis*	Y-23^T	CP098732.1	84.17	28.3	90.78
80	*A.tjernbergiae*	DSM14971^T	ARFU00000000.1	74.66	21.3	76.44
81	*A.towneri*	CIP107472^T	APPY00000000.1	76.48	21.5	79.80
82	*A.ursingii*	CIP107286^T	APQA00000000.1	74.43	21.1	76.67
83	*A.variabilis*	NIPH2171^T	APRS00000000.1	75.15	21.0	78.14
84	*A.venetianus*	CIP110063^T	APPO00000000.1	74.58	21.6	76.96
85	*A.vivianii*	NIPH2168^T	APRW00000000.1	74.49	21.3	76.55
86	*A.wanghuae*	dk386^T	CP045650.1	75.45	21.2	77.76
87	*A.wuhouensis*	WCHA60^T	CP031716.1	75.17	21.6	76.45
88*	*Acinetobacter* sp.	ANC4910	SJNT01000001:24	83.89	27.8	90.96

* The closest match strain obtained by screening full-length 16S rRNA sequences in NCBI databases.

Table S2 The list of all negative traits of strain through the test of commercial kits

The name of commercial kits	Negative traits of strain A7.4^{T}
API 20 NE	Nitrate reduction, Tryptophan Deaminase, Glucose Fermentation, Arginine Dihydrolase, Urease, Gelatin Hydrolysis, β-Galactosidase, Glucose assimilation, Arabinose assimilation, Mannose assimilation, Mannitol assimilation, N-Acetyl glucosamine assimilation, Maltose assimilation, Gluconate assimilation, Caprate assimilation, Adipate assimilation.
API 50CH	Glycerol, Erythritol, D-arabinose, L-arabinose, D-ribose, D-xylose, L-xylose, Adonitol, β-methyl-D-xyloside, D-galactose, D-glucose, D-fructose, D-mannose, L-sorbose, Rhamnose, Dulcitol, Inositol, Mannitol, Sorbitol, α-methyl-D-mannoside, α-methyl-D-glucoside, N-acetylglucosamine, Amygdalin, Arbutin, Esculin ferric citrate, Salicin, D-cellobiose, D-maltose, D-lactose, D-melibiose, D-sucrose, D-trehalose, Inulin, D-melezitose, D-raffinose, Starch, Glycogen, Xylitol, Gentiobiose, D-turanose, D-lyxose, D-tagatose, D-fucose, L-fucose, D-arabinol, L-arabinol, Potassium gluconate, Potassium 2-ketogluconate, Potassium 5-ketogluconate, 2-Keto-gluconate.

Table S3 Shared carbon source assimilation patterns between strain A7.4^T and reference strains.

Carbon source	1	2	3	4
Acetate	+	+	+	+
Adipate	-	-	-	-
4-Aminobutyrate	+	+	+	+
L-Arabinose	-	-	-	-
L-Arginine	+	+	+	+
L-Aspartate	+	+	+	+
Azelate	-	-	-	-
Benzoate	+	+	+	+
Citraconate	-	-	-	-
Citrate	+	+	+	+
Ethanol	+	+	+	+
Gentisate	-	-	-	-
D-Gluconate	-	-	-	-
D-Glucose	-	-	-	-
L-Glutamate	+	+	+	+
Histamine	-	-	-	-
L-Histidine	+	+	+	+
4-Hydroxybenzoate	+	+	+	+
DL-Lactate	+	+	+	+
Levulinate	-	-	-	-
D-Malate	+	+	+	+
Malonate	+	+	+	+
L-Ornithine	+	+	+	+
Phenylacetate	+	+	+	+
L-Phenylalanine	+	+	+	+
D-Ribose	-	-	-	-
Trigonelline	-	-	-	-

Strains: 1, *Acinetobacter zhairuonense* A7.4^T; 2, *Acinetobacter tibetensis* Y-23^T; 3, *Acinetobacter tandoii* CIP107469^T; 4, *Acinetobacter kanungonis* PS-1^T.

+, Positive; -, negative.

Assimilation data were provided by Professor Alexandr Nemec (National Institute of Public Health, Prague, Czech Republic).

Table S4 Cellular fatty acid compositions of strain A7.4^T and reference strains.

Fatty acid	1	2	3	4
$C_{10:0}$	0.8	2.5	1.18	
$C_{12:0}$	7.5	7.2	8.1	7.3
$C_{12:0}$ 2-OH	1.5	–	2.5	2.3
$C_{12:0}$ 3-OH	5.1	8.5	6.5	6.7
$C_{16:0}$	**16.7**	8.9	**15.6**	**15.8**
$C_{17:0}$	0.3	2.4	2.0	3.4
$C_{17:1}$ ω8c	1.4	4.5	1.9	2.6
$C_{18:0}$	1.5	3.6	2.2	2.9
$C_{18:1}$ ω7c	3.0	3.6	2.3	2.1
$C_{18:1}$ ω9c	**22.2**	**35.5**	**24.6**	**30.6**
Summed feature 3 ($C_{16:1}$ ω7c/$C_{16:1}$ ω6c)	**31.2**	**20.1**	**24.3**	**19.5**

Strains: 1, *Acinetobacter zhairuonense* A7.4^T; 2, *Acinetobacter tibetensis* Y-23^T; 3, *Acinetobacter tandoii* CIP107469^T; 4, *Acinetobacter kanungonis* PS-1^T.

Note: Values are percentages of the total fatty acids. The major fatty acids (greater than 10.0 %) are shown bold. The trace fatty acids (< 0.5%) are shown " – ".
